# Metronomic chemotherapy and anti-angiogenesis: can upgraded pre-clinical assays improve clinical trials aimed at controlling tumor growth?

**DOI:** 10.1111/apm.12201

**Published:** 2013-10-26

**Authors:** Klas Norrby

**Affiliations:** 1 Department of Pathology, Institute of Biomedicine, Sahlgrenska Academy, University of GothenburgGothenburg, Sweden

**Keywords:** Metronomic chemotherapy, angiogenesis assays, *in vitro*, *in vivo*, autochthonous tumors, reactive oxygen species, inflammation

## Abstract

Metronomic chemotherapy, which is continuously administered systemically at close to non-toxic doses, targets the endothelial cells (ECs) that are proliferating during tumor angiogenesis. This leads to harmful effects of an even greatly increased number contiguous tumor cells. Although pre-clinical studies of angiogenesis-related EC features *in vitro* and of the anti-angiogenic and anti-tumor effects *in vivo* of metronomic chemotherapy have provided valuable insights, clinical trials with this type of therapy have been less successful in inhibiting tumor growth. One possible reason for the apparent disconnect between the pre-clinical and clinical outcomes is that most of the currently used experimental angiogenesis assays and tumor models are incapable of yielding data that can be translated readily into the clinical setting. Many of the assays used suffer from unintentional artifactual effects, e.g., oxidative stress *in vitro*, and inflammation *in vivo*, which reduces the sensitivity and discriminatory power of the assays. Co-treatment with an antioxidant or the inclusion of antioxidants in the vehicle often significantly affects the angiogenesis-modulating outcome of metronomic mono-chemotherapy *in vivo*. This ‘metronomic chemotherapy vehicle factor’ merits further study, as do the observations of antagonistic effects following metronomic treatment with a combination of standard chemotherapeutic drugs *in vivo*.

The major obstacles to devising effective treatments for cancer are the heterogeneity and genetic instability of tumors, as a heterogeneous population of tumor cells contains many genetic and epigenetic variations. Tumor growth and spread are angiogenesis-dependent processes, which makes the endothelial cells (EC) in tumor vasculature a desirable target for anti-tumor agents. Metronomic chemotherapy is a promising treatment modality, in that it primarily targets the proliferating microvascular ECs that participate in tumor angiogenesis. The basis for this therapy is the frequent administration of low, close to non-toxic doses of cytotoxic agents. For several cytotoxic agents, metronomic chemotherapy has been shown to suppress angiogenesis and tumor growth in pre-clinical studies without causing severe side effects, which are often problematic in conventional chemotherapy. Thus, metronomic chemotherapy has the potential to improve considerably the quality of life of cancer patients.

The results of clinical trials conducted to date with metronomic chemotherapy do not satisfactorily match the expectations raised by the compelling pre-clinical data, obtained for this regimen. One may assume that the better the pre-clinical data are from a biological point of view, the higher the probability that their clinical application will improve the outcome.

Pre-clinical studies on the effect of metronomic chemotherapy on angiogenesis are performed *in vitro*,* ex vivo,* and *in vivo*. *In vitro*, cultured macro- or micro-vascular ECs, as well as tumor cells, are used. Studies conducted *in vivo* involve a variety of assays in tumor-free tissues and also look at the effects of low-dosage chemotherapy on tumor growth. The present paper discusses certain aspects of currently used pre-clinical models encompassing cell culture, angiogenesis assays, and tumors in the perspective of metronomic chemotherapy.

## Metronomic Chemotherapy and Angiogenesis

Chemotherapy exerts anti-mitotic and anti-proliferative activities on dividing cells, primarily by increasing the intracellular production of reactive oxygen species (ROS) rather than through discrete pharmacologic mechanisms, as discussed previously 1,2. In 2000, the laboratories of Folkman and Kerbel showed that by altering the dosing regimen to one of regular inoculations without rest periods using low, minimally toxic concentrations of the drugs, which is known as ‘metronomic’ chemotherapy dosing, certain commonly used cytotoxic drugs produced anti-angiogenic effects in xenotransplant models, even against drug-resistant tumors 3–5. This type of treatment schedule differs from conventional chemotherapy, which uses the maximum tolerated dosages of drugs (directed against rapidly dividing tumor cells) and which through targeting of normal tissues and cells causes toxic effects in the patient and makes it necessary to have a resting period of ˜3 weeks per treatment cycle. During the resting periods, the damaged microvasculature can recover, resulting in an insufficient anti-angiogenic effect over the whole treatment cycle of conventional chemotherapy.

### How normal are the endothelial cells and the vasculature in tumors?

Although tumor-associated angiogenesis has traditionally been defined as the sprouting of new microvessels from pre-existing microvessels, it is maintained that the blood vessels that support tumor growth or tumor rebound from therapy-induced trauma may originate from cells that are recruited from the bone marrow or that differentiate from tumor stem cells, in a process termed ‘vascular mimicry’ 6. However, considerable controversy surrounds the nature and function of bone marrow-derived circulating EC progentitors 7,8. Furthermore, the tumor vasculature may, in many instances, contain cytogenetically abnormal ECs, which may even harbor tumor-specific genetic material. Although the biological features of these genetically abnormal EC-like cells have not been fully elucidated, it is clear that these cells can mutate to acquire drug resistance and generate drug-resistant populations more readily than normal ECs 9–14. Nevertheless, the phenotypes of genetically abnormal tumor-associated ECs are reported to be highly stable both *in vitro*, in human xenografts, and following serial *in vivo* passage 15.

### It is not only the vascular endothelial cells in tumors that are affected by metronomic chemotherapy

In general, tumors are made up of neoplastic cells, ECs, smooth muscle pericytes, other perivascular cells, stromal cells, and various inflammatory cells. In a hypoxic environment, which is characteristic of most tumors, all these cell types are able to produce and release pro-angiogenic (and proliferative) factors, including vascular endothelial growth factor-A (VEGF-A), platelet derived growth factor, acid fibroblast growth factor, base fibroblast growth factor (bFGF), angiopoietins, and stromal cell-derived factor 1 6,16,17. Moreover, tumor-associated macrophages, mast cells (MCs), and neutrophils are recruited and generate proliferative signals that act on fibroblasts and perivascular cells, as well as pro-angiogenic signals, all of which affect the extracellular matrix. Although ECs and platelets are exposed extensively to systemically administered drugs, all the other intra-tumoral and extra-tumoral proliferating cells in the body are affected to various extents by metronomic chemotherapy.

A complicating factor in these studies is that there is accumulating evidence to suggest that the efficacy of metronomic chemotherapy does not rely exclusively on its anti-angiogenic activities (via direct effects on ECs). Several complementary activities, such as the restoration of various anti-cancer immune responses and the induction of tumor dormancy, have been described for metronomic chemotherapy 11. Metronomic chemotherapy can also stimulate the production of endogenous anti-angiogenic factors. One example is thrombospondin, which is produced in diverse cells, including platelets, and acts as a potent and EC-specific inhibitor of angiogenesis 18–20.

As noted above, metronomic chemotherapy is now considered as a form of multitargeted therapy, which can impose long-term adverse effects, including a high incidence of secondary leukemia in children and young adults 21.

### Moderately successful clinical outcomes for metronomic chemotherapy

Pre-clinical data have confirmed anti-angiogenic and/or anti-tumor effects for metronomic chemotherapy with several cytotoxic agents. Numerous clinical trials have been performed in which metronomic chemotherapies, often using a combination of two or more cytotoxic agents and including specific anti-angiogenic agents such as anti-VEGF-A antibodies, have been tested 21–24.

Even though promising results have emerged from a number of clinical trials, it is generally considered that metronomic chemotherapy has not lived up to the expectations raised by the promising anti-angiogenic and anti-tumor pre-clinical testing. In several cases, these agents have proved disappointing in phase II/III clinical studies 11,25–28. This lack of consistency between the pre-clinical and clinical results raises questions as to whether the current pre-clinical angiogenesis and tumor models are adequate in providing useful data for clinical applications concerning metronomic chemotherapy.

One of the main limitations in this field is the current lack of relevant and reliable biomarkers (i.e., diagnostic, predictive, and surrogate markers) to enable identification of those patients who are most likely to benefit from metronomic chemotherapy 21. Furthermore, it appears that a single metronomic regimen is unlikely to have universal efficacy; the optimal combination regimens for metronomic chemotherapy remain to be determined for any given tumor type in patients 11,21.

### Drug-specific and unexpected effects of drug combinations *in vivo*

In dose–response experiments, several metronomically administered standard chemotherapeutic drugs significantly suppress angiogenesis, while other drugs show no effect and several drugs significantly stimulate VEGF-A-mediated angiogenesis, as observed using one and the same *in vivo* model, i.e., the tumor-free rat mesentery assay 29–31. In fact, low-dosage metronomic monotherapy can have dramatically different outcomes depending on the drug used: paclitaxel, vinblastine, and cyclophosphamide have anti-angiogenic effects; doxorubicin and epirubicin exert no effects on angiogenesis; and cisplatin, 5-fluorouracil, irinotecan, and mitoxantrone stimulate angiogenesis 29–33. Even at higher doses, using bolus scheduling, significant and distinct drug-specific angiogenesis-modulating effects are observed 34,35. Clearly, there are dose-related effects with these regimens.

It is noteworthy that the angiogenesis-modulating effects of many metronomically administered drugs are significantly influenced by antioxidants, given either as a co-treatment or present as components of the vehicle, resulting in either reduction or enhancement of the drug-specific effect 29,32. Therefore, there is a ‘metronomic chemotherapy vehicle factor’, which is related to the presence of antioxidants and the redox balance with the potential to affect significantly the outcome, as first observed by Albertsson et al. 29.

The finding that the administration of low-dosage 5-fluorouracil or cisplatin promoted VEGF-A-mediated angiogenesis, an effect that was not observed when 5-fluorouracil or cisplatin compounds were administered at higher doses 30,31, might be attributed to the fact that angiogenic response to 5-fluorouracil or cisplatin is Bell-shaped 27. An alternative explanation is that the pro-angiogenic effect of metronomic mono-chemotherapy with irinotecan, mitoxantrone, cisplatin, or 5-fluorouracil is due mainly to a moderate increase in intracellular ROS, which triggers angiogenic responses through the production of pro-angiogenic factors, including VEGF-A, in the targeted ECs (and possibly other cells), as discussed below. It could alternatively relate to the drug-specific effects on platelets, which are able to release either pro- or antiangiogenic factors, as discussed below.

The original suggestion 3–5 that metronomic scheduling of almost any standard chemotherapeutic drug should suppress angiogenesis is also called into question by the finding that treatment combinations may strongly influence the efficacies of individual cytotoxic agents. In a xenograft tumor model in mice, metronomic irinotecan monotherapy suppressed tumor growth and tumor vascularity, whereas metronomic monotherapy with 5-fluorouracil or oxaliplatin had no effect on the tumor 36. Moreover, metronomic therapy with irinotecan, 5-fluorouracil, and oxaliplatin in combination had no effect on tumor vascularity 36. In a study that used the rat mesentery assay, epirubicin monotherapy did not influence VEGF-A-induced angiogenesis, whereas monotherapy with the low-molecular-weight heparin dalteparin (an anti-coagulant and antioxidant) acted to promote angiogenesis; interestingly, the treatment with a combination of these two agents significantly inhibited angiogenesis 33. Thus, combination regimens can yield surprising outcomes and they may not always be more effective than single-drug therapy 36. A possible explanation of these findings is that the combined effects of chemotherapy on individual signaling pathways are additive, synergistic, or antagonistic, and depend not only on the oxidative anti-tumor agent/cytotoxic drug examined but also the dosages employed 1.

The extent of which these chemotherapeutic agents with or without pro-angiogenic effects pertain to tumor angiogenesis is difficult to corroborate because, as noted above, several anti-tumor modes unrelated to angiogenesis may operate in parallel during metronomic chemotherapy 21,32.

## Cell Culture

*In vitro* angiogenesis assays are useful for screening potential targets and provide an early validation step in the process of testing a new drug, owing to their rapid implementation and ease of quantitation. Extracellular matrix substitutes, such as collagen gel and Matrigel, are popular components of *in vitro* 3-D angiogenesis assays because they enable tubule formation by cultured ECs. However, these assays are usually used with a single cell type, which lacks the complex multicellular interactions that are essential for angiogenesis.

### Cell selection, phenotypic alteration, and oxidative stress

Cell culture studies are used to investigate the molecular effects and mechanisms associated with ECs or tumor cells following exposure to low doses of a chemotherapeutic agent(s) in the growth medium. It should be remembered that the establishment of an EC line necessarily entails an initial selection of the EC population from the site of isolation, with additional rounds of selection occurring during subsequent subculturing. It should also be noted that ECs, as is the case for all cells, undergo phenotypic alterations *in vitro* when incubated in media that contain growth factors and other components, to which the cells adapt.

Multiple enzymes that use molecular oxygen as a substrate generate ROS. In cells, there is a fluctuating redox balance between the effects of ROS and enzymatic or non-enzymatic antioxidant systems. Various non-enzymatic molecules (e.g., glutathione, vitamins A, C, and E, and flavonoids), as well as enzymatic scavengers of ROS (e.g., superoxide dismutase, catalase, and glutathione peroxide) act to reduce or balance the intracellular levels of ROS 1.

For conventional culturing techniques, there is an increase in the level of ROS because the O_2_ pressure is dramatically increased: normal culture conditions are basically a state of hyperoxia. Most cells in the human body are exposed to O_2_ pressure in the range of 1–10 mmHg, whereas for conventional culture conditions with 95% air and 5% CO_2_, the O_2_ pressure is dramatically increased to 150 mmHg 37.

The largest proportion of intracellular ROS production occurs in the mitochondria 38,39. Various exogenous agents, including chemotherapy, trigger intracellular ROS generation, thereby disrupting the cellular redox balance homeostasis with consequences for cellular functions, as ROS act as intracellular mediators (see below). An excess of ROS causes oxidative stress, and a high level of oxidative stress harms cells. Accumulation of ROS within cells and/or their release into the culture medium is highly specific for the cell type being examined 40.

### Culture medium-specific effects on ROS production

In general, basic cell growth media are deficient in antioxidants. The amount of ROS produced during growth in culture medium depends not only on the cell type but also on the composition of the medium and whether the culture is incubated in light or (to a lesser extent) in the dark 41–43. The buffering capacity and composition of the medium are important with regard to the behavior of the cultured cells and may affect significantly the results 44. In fact, it has been suggested that cell studies using different cell growth media are scarcely comparable 43.

### ROS act as intracellular mediators

At physiologically low or moderate levels, ROS act as signaling molecules in essential metabolic pathways, including the induction of gene expression 1,45 of various growth factors, including VEGF-A, which is a key pro-angiogenic and cell survival factor 45–50. Notably, the combined effects of individual metabolic pathways can be additive, synergistic, or antagonistic, and depend not only on the oxidative agent/anti-tumor agent examined but also the dose employed and the cell type in which they are analyzed, which might explain the pre-clinical finding of antagonism in relation to angiogenesis reported by Fioravanti et al. 36, as discussed above.

Typically, low levels of ROS, particularly those of hydrogen peroxide (H_2_O_2_), promote cellular proliferation, whereas ROS levels reduced with the aid of potent antioxidants to below the homeostatic set point may inhibit the physiologic role of oxidants in cellular proliferation 1,39. ROS at high levels have a propensity to push the cell to the brink of toxicity and even to eventual apoptosis and cell death 51,52.

### Cell types and variables that are commonly studied in relation to angiogenesis

In cultures of ECs, the variables of cell proliferation, differentiation, migration, tube formation, growth factor production, altered gene expression, and apoptosis are commonly studied. The interactions of ECs with the surrounding supporting cells, such as fibroblasts, and extracellular matrix substitutes have been studied 28. The microvascular EC is often the preferred cell type for these studies. However, it should be remembered that there is genetic heterogeneity among the microvasculature across different sites 53, which may complicate comparisons between different microvascular cell lines.

Cultured tumor cells are used to investigate proliferation and the extent to which low doses of chemotherapeutic agents affect the receptor repertoire and production of growth factors, particularly VEGF-A. Moreover, the results of such experiments are often uncertain as tumor cells basically have markedly increased oxidative stress levels compared with tumor cells usually seen *in vivo* 39,54.

### Can the *in vitro* procedures be improved?

Modification in simple experimental variables, such as the choice of medium buffer, the control of exposure to light, the pH of the medium, and the amount of ROS in the medium 40,43, as well as oxygen-controlled cultures 55 could arguably improve the outcomes from a biological point of view. If all of these factors were controlled, the results obtained from *in vitro* studies on angiogenesis would be more reproducible and thereby more useful for the design and interpretation of animal experiments and clinical trials.

## *Ex Vivo* Assays (Organ Culture)

The *ex vivo* assays are all similar in that segments, disks, or sections of a specific tissue type are cultured in a 3-D matrix *in vitro* and are monitored for microvessel outgrowth over a period that can extend up to a couple of weeks 28. A major problem with all these assays is that they employ non-human tissues, which raises questions as to their applicability as pre-clinical screening assays, given that the responses to various drugs or test substances may be species-specific 28.

Although not a traditional *ex vivo* assay, the exteriorized rat mesentery has been used for real-time observations of the mesentery; this model has proven to be extremely useful in identifying novel cellular events in angiogenesis 56,57.

## Inflammation Interferes with Angiogenesis and Anti-angiogenesis Responses to Drugs *in vivo*

Angiogenesis is a hallmark of both hypoxia and inflammation. Inflammation occurs in various forms in response to different host tissue injuries, such as hypoxia, tissue trauma (including implantation of foreign material) that induces wound healing reactions, infections, and tumor development 58. In reaction to tissue injury, ROS are generated and a multifactorial network of chemical signals initiates and maintains host responses that are designed to ‘heal’ the affected tissue. This process involves the activation and directed migration of inflammatory cells, which include neutrophils, monocytes, and eosinophils, from the venous system to the sites of damage; monocytes differentiate into macrophages after extravasation from the circulation into tissues.

Among the innate immune cells, macrophages, MCs, and dendritic cells (DCs) serve as sentinel cells, residing in tissues and continuously monitoring the cellular microenvironment for signs of distress. When tissue homeostasis is disturbed, the sentinel cells release soluble mediators that act singly or in combination to induce the mobilization and infiltration of neutrophils and other leukocytes into the damaged tissue. Macrophages and MCs also induce metabolic and proliferative fibroblast responses and are able to initiate and sustain potent angiogenic activities 59–63. Once activated, macrophages are considered to be the main source of growth factors and cytokines, which exert profound effects on ECs and other cells in the local microenvironment. Plasmacytoid DCs are able to support angiogenesis 64, whereas tumor angiogenesis is reported to depend largely on the activities of immature DCs 65. In certain tumors, MCs are essential for the development of angiogenesis 66,67. MC-mediated angiogenesis was discovered using the rat mesentery angiogenesis assay 61,68.

In phylogenetic terms, the MC represents an ancient cell type found in all species that have a blood circulatory system, and it precedes lymphocytes and other cells of the immune system 69. It has been suggested that the MC is essential for promoting and orchestrating inflammation 69, so it may also be involved in orchestrating inflammation-induced angiogenesis. MCs are important cells owing to their release of stored and newly synthesized inflammatory mediators and growth factors, which include histamine (and serotonin in certain species), cytokines, such as VEGF, bFGF, IL-1, IL-6, IL-8, and TNF, proteases, lipid mediators, and heparin, many of which can individually promote angiogenesis 61,66,70.

Clearly, using inflammatory activity-inducing experimental procedures, the effect of any given test agent or treatment regimen is distorted to an unknown degree by the artifactually induced angiogenesis. Unfortunately, most mammalian *in vivo* angiogenesis assays employ procedures that cause cellular damage to the test tissue, thereby initiating some level of non-specific angiogenesis, which diminishes the sensitivity of the assay.

## Analysis of Anti-angiogenesis in Tumors *In Vivo* is Particularly Challenging

To date, no method has been developed that accurately assesses the anti-angiogenic effect *per se* in a tumor, as inhibition of angiogenesis limits tumor growth and vice versa, which is a major impediment to the clinical development of many anti-angiogenic drugs. Furthermore, there is a severe lack of reliable biological surrogate markers of anti-angiogenic effects in tumors. Assessing angiogenesis in cancer patients is currently limited to the use of functional measures derived using various imaging modalities 6,71.

An often-used method for quantifying tumor vascularity, and purportedly angiogenesis, involves the measurement of microvessel density (MVD) based on immunohistochemical visualization of EC epitopes, such as CD34 or CD105, in excised tissue samples or in superficially located tumors. Although tumor MVD analysis of biopsies is a useful prognostic indicator for patients with most types of cancer, it is not suitable for measuring angiogenic activity or assessing the angiogenic dependence of a tumor 72. Therefore, the possibilities to assess angiogenesis and anti-angiogenic effects in patients are currently very limited.

Rodent studies are vital for the development of novel anti-cancer therapeutics and are used in pharmacokinetic, pharmacodynamic, toxicology, and efficacy studies. The mouse is undoubtedly the most widely used species in these experiments, primarily because various inbred and genetically engineered or manipulated strains are available.

### Syngeneic mouse tumor models

Tumor cell lines, which are propagated in culture for a long period of time before being injected into the mouse, are frequently used. As a rule, these tumors grow very rapidly and are highly aneuploid, which makes them quite dissimilar to the types of tumors generally seen in clinical practice.

### Xenograft tumors

Using immunocompromised animals, non-syngeneic tumor cells are injected as a bolus into a tissue that is disrupted and disorganized by the ectopic transplantation of a high number of foreign cells. As the tumor transplant may initially be severely hypoxic, angiogenesis is highly dependent on expression of the Id gene, which exerts a selection pressure on the tumor cell population. When human tumor cells or tumor tissue pieces are implanted into immunocompromised animals, the developing xenograft tumor stromal cells, extracellular matrix, blood components, sentinel immune cells, and any EC progenitors recruited from the bone marrow all originate from the host. The tumor endothelium is probably mainly of host origin.

### Orthotopic tumors

Implantation of cultured tumor cells into the organ of origin is thought to allow organotypic interactions between the tumor cells and surrounding stroma. Intratumoral lymphangiogenesis is absent from orthotopic and xenograft tumors, which may affect experimental end-points, such as the occurrence/timing of metastasis, tumor progression, and survival.

### Autochthonous tumors

Autochthonous tumors originate in the place where they are found and feature a physiologic stroma–tumor relationship, as in human cancer. There are major differences in angiogenesis between transplanted or xenograft tumors and autochthonous tumors 25,66,73. The usefulness of xenograft models for efficacy testing has been questioned, whereas tumors in genetically engineered mouse models (GEMMs) may offer advantages with respect to efficacy assessments 74,75, as these latter tumors arise autochthonously. GEMM tumors occur in animals that have an intact immune system and unperturbed DNA repair mechanisms. Moreover, differences in the gene expression of tumor-associated immune sentinel cells, such as macrophages, between xenografts and GEMMs have been observed 75.

### Metastasis

Tumor cell spread to and tumor growth at secondary sites are extremely important clinical events, as the majority of cancer-related mortality is associated with metastatic tumors, rather than the primary tumor. Unfortunately, there is a lack of reliable pre-clinical metastasis models, although in many orthotopically implanted models, metastasis occurs but it is very heterogeneous and not detectable in all animals after implantation. Metastasis models involving autochthonous tumors are eagerly awaited.

### Mouse strain-specific characteristics

In addition to species-specific features, strain-specific differences with respect to various features that influence angiogenesis have been reported in mice, which can complicate comparisons between experiments. There are significant differences in the responses to pro-angiogenic factors among inbred mouse strains 76. There is a striking correlation between highly genetically heterogeneous bFGF- or VEGF-A-induced angiogenesis and the intrinsic levels of circulating EC progenitors among different inbred mouse strains 7. In addition, the genetic backgrounds of inbred strains determine the inflammatory-induced angiogenesis responses in these mice 77,78. Similar strain-specific differences can be expected in other species.

## Comparing and Evaluating Pre-clinical *In Vivo* Angiogenesis Assays in Terms of their Usefulness in Guiding Clinical Trials

The key to generating accurate dose–response curves is having knowledge of the release rate and the spatial and temporal concentration distributions of the exogenously administered pro-angiogenic or anti-angiogenic test agent(s). However, this is very difficult to control in any of the currently used models. A complicating fact is that the dose–responses can be Bell-shaped or U-shaped, as discussed elsewhere 27. Thus, unambiguous dose-effect studies are very rare, if they exist at all, and stringently controlled dose-effect studies that compare two or more assays in parallel have not been published to our knowledge. Each experimental approach offers specific advantages and suffers from certain limitations.

### Administration routes and scheduling of test agents

The simplest way to administer a test agent is via the oral route, whether this is performed experimentally or in the clinical setting. If oral delivery works, it becomes the favored route. However, the injection of test agents subcutaneously, intravascularly, or intraperitoneally is more common. Through injection, a controlled rate and dosage of the test solution can be achieved over a relatively long time period per setting; osmotic minipumps that release test solution at a constant rate are used extensively in mice and rats. Dosing scheduling of chemotherapeutics can thus vary considerably across experiments. The perfect scheduling regimen for each test agent relates to its toxicity, pharmacokinetic, and pharmacodynamic properties. Conclusive studies on the influence of scheduling on angiogenesis-modulating effects of metronomic chemotherapy remain to be performed.

### Experimentally related disarray of the model

The impact of unintentional inflammation-induced angiogenesis, which may confound the results of metronomic chemotherapy, should be minimized or at least taken into account when the data are interpreted. Furthermore, disruption of the microvascular basal membranes during tissue trauma is a pro-angiogenic cue. The toxic effects of drugs should be kept under strict control, as toxicity may mask specific angiogenesis-modulating drug effects. However, toxic effects in terms of retarded or decreased body weight are easily overlooked in species that exhibit very slow physiologic growth during adulthood (e.g., mice), whereas these effects are more noticeable in rats, which exhibit robust physiologic growth in adulthood.

### Data acquisition and verification

It is important to be able to assess the combined effect on angiogenesis of two or more drugs that are administered simultaneously or sequentially systemically, not only for the evaluation of scheduling of combination therapies but also because strong antagonism may emerge from metronomic chemotherapy 36. For molecular-structure analyses, truly quantitative and sensitive assays are required. The value of data obtained in one species is greatly increased if the data are confirmed in a second mammalian species. In this regard, the current predominant use of a single species (i.e., mice) in angiogenesis studies is a matter of concern.

### Use of vascularized test tissues to take into account effects of circulating cells and platelets

A major obstacle to the progress of research on angiogenesis *in vivo* is that measurements of neoangiogenesis are confounded by the presence of well-developed vascular networks in virtually all mammalian tissues. Nevertheless, assays of vascularized tissues are needed to take into account the contributions of platelets and other circulating cells, including EC precursors from the bone marrow 7, which promote angiogenesis. Platelets accumulate angiogenesis-regulatory proteins in two sets of alpha-granules, with positive regulators in one set and negative regulators in the other set, which may be released separately 79,80, depending on the type of therapeutic molecule used. These two populations of alpha-granules are distinct in terms of pharmacologic and morphologic features 79. The overall effect on tumors of platelet–endothelium interactions is thought to stimulate tumor angiogenesis 81.

## *In Vivo* Angiogenesis Assays

Is it conceivable that tumor-free models can provide pertinent information regarding tumor anti-angiogenic and tumor-suppressing effects in the clinical setting?

Almost all current assays are based on the analysis of tumor-free tissues. As the inhibition of angiogenesis limits tumor growth and vice versa, it can be argued that studies on the angiogenesis-modulating effects of any potent anti-tumor therapy should be conducted in a tumor-free tissue. It is notable that the ECs in the human tumor vasculature have a gene expression pattern that resembles that of angiogenically activated ECs in normal human tissues 82.

*In vivo* angiogenesis reflects the complex, stepwise, multifactorial, multicellular process that leads to the formation of new blood microvessels, which cannot be mimicked by *in vitro* studies. Although there are numerous *in vivo* angiogenesis assays and models, their relevance from a clinical perspective remains to be demonstrated unequivocally; thus, no currently used *in vivo* assay is optimal. The merits and limitations of the current *in vivo* angiogenesis assays have been reviewed repeatedly 28,70,83,84, and it would appear that the criteria listed in Table1 define high-quality assays from the biological standpoint.

**Table 1:** Desired features of biologically appropriate *in vivo* angiogenesis assays

Mammalian-based, so as to capture responses to mammalian proteins 100
Natively vascularized, to incorporate the effects of circulating endothelial progenitor cells and platelets
No significant angiogenesis occurring in the adult test tissue if *de novo* angiogenesis is to be studied
Displaying minimal non-specific, artifactually induced inflammation, as inflammation induces angiogenesis, which may interfere with the results
Truly quantitative in terms of microvessel formation, which is a prerequisite for molecular-activity and dose-effect studies, although analyses of dose-effect responses in a strict sense are probably not yet achievable, as discussed in the text
Allowing the recording of concurrent influences of two or more test agents administered systemically in parallel or sequentially, given that antagonistic or unforeseeable effects may occur 33,36
Test animals should have mature liver function for metabolizing drugs
Any significant toxic influence of the treatment should be measurable
High-quality animal facilities and experimental procedures are highly recommended
Ethically acceptable

In Table2, some examples from the literature are given of important mammalian models, as well as the embryonic avian chorioallantoic membrane (CAM) assay. The microsurgical corneal, CAM, and Matrigel assays are probably the most widely used angiogenesis models, and the results obtained using these models have been extensively tested in clinical trials. There is also an interesting model, which is adapted from the Matrigel plug assay, in which human blood-derived endothelial progenitor cells and mature smooth muscle cells are delivered subcutaneously into immunodeficient mice, resulting in the *de novo* formation of a vascular network and the development of functional anastomoses with the host circulatory system 85.

**Table 2:** Critical synopsis of a selection of the main currently used mammalian *in vivo* angiogenesis assays, as well as the chick chorioallantoic membrane (CAM) assay, listed in chronologic order of their introduction

Assay	Advantage(s)	Disadvantage(s)
CAM	Technically simple to conduct both in ovo and *in vitro*	Avian embryonic tissue, in which all tissue cells proliferate
	Test agent applied via a carrier onto the CAM surface	Sometimes difficult to distinguish angiogenesis from artifactual increases in blood vessel density linked to tissue contraction caused by the applied carrier
	Suitable for large-scale screening experiments	
	Permits non-invasive observations	Very sensitive to increases in oxygen tension; production of ROS
	Permits biochemical and genetic analyses^4^	Sprouting angiogenesis is followed by intussusceptive microvessel growth
	Suitable for mammalian xenografts until EDD 15–18	Pro-angiogenic treatment accelerates and anti-angiogenic treatment suppresses constant organogenic angiogenesis
	Inexpensive	Induction of (*de novo*) angiogenesis is possible only after EDD 12–13
	Drugs can be administered topically onto the CAM, injected i.v. (difficult), injected i.p. into the body of the embryo or injected into the yolk sac and amnion	The tissue is overly sensitive to inflammatory angiogenesis
	No ethical issues	Drugs that require metabolic activation cannot be assessed due to liver immaturity
	Lack of excretion from the CAM allows test agents to be maintained in the circulation for extended periods	The relevance to human angiogenic diseases is limited; non-mammalian species, which may respond differently to mammalian proteins 100
Corneal micropocket^5^, ^6^	New vessels, except the smallest microvessels, are, in principle, easily identifiable	Atypical angiogenesis, as the normal cornea, is avascular because of the trapping of soluble VEGFR-1
	Mammalian model: mice, rats, and rabbits are used	Technically demanding, especially in animals with small eyes, as in mice
	Permits non-invasive observation	The surgery- or suture-induced lesion causes inflammation and angiogenesis
	Quantitative assessment is reported	Toxicity within the micropocket area is difficult to assess
		Expensive
	Immunologically privileged site before vascularization, allowing tumor implantation	Ethically questionable as the cornea is a sensory organ
		The cornea is not a highly relevant site for tumor growth
	Angiogenesis by sprouting	Only a few substances can be tested in one setting
		Exposure to oxygen via the corneal surface causes angiogenesis through the generation of ROS
Mesentery^4^, ^5^	Mammalian adult tissue; natively vascularized; lacks significant physiologic angiogenesis	Time-consuming, especially when assessing the numbers and lengths of individual microvessel segments and sprouts
	The test tissue is visceral; visceral organs are common sites of primary cancers and metastasis	Does not allow real-time observations^9^
	Minimal trauma, if any, is inflicted upon the test tissue by i.p. injection of the test solution	Mice are much less suitable than rats for quantitative angiogenesis
	Truly quantitative, allowing dose–response and molecular-activity studies^7^ with respect to spatial extension, density, length of individual microvessel segments, and sprouts *in sit*u^8^	Rats demand 10 times higher levels of test agents than mice
	Suitable for measurements of growth factor-induced signaling	Relatively few substances and doses can be tested in one setting
	Sprouting angiogenesis, which predominates in normal tissues and tumors	The mesentery is very sensitive to endotoxin, which induces VEGF expression and angiogenesis (endotoxin-free solutions should be used for i.p. injection) 105,106
	Toxicity data easily acquired in rats, which grow robustly physiologically in adulthood	Intra-abdominal surgery rapidly disturbs homeostasis causing angiogenesis^9^
		Less suitable for screening
	Allows for the two-dimensional visualization of entire microvascular networks down to single cell level	Few genetically engineered rat strains are available
	Allows for testing of multiple agents simultaneously or sequentially	Technically fairly demanding
Matrigel plug^5^, ^6^	Technically simple when used s.c.	Matrigel is not chemically defined and contains growth factors (even in the growth factor-reduced form)
	Rapid quantitative analysis assessing vascular-specific tissues in chambers with 3-D defined plugs	Difficult to make plugs in a uniform 3-D shape (except in chambers)
		No angiogenic response to VEGF in chambers
	Suitable for large-scale screening	Analysis of plugs is time-consuming for tissues other than vascular-specific tissues
		Does not allow real-time observations
		The s.c. tissue is not highly relevant for tumor growth
		Plugs lack cells that are able to produce endogenous pro- and anti-angiogenic factors, which would affect vascular responses
		Expensive

Compiled from the following references: 28,70,83,84.

EDD, Embryonic development day; s.c., subcutaneous; i.p., intraperitoneal; i.v., intravascular; VEGF, vascular endothelial growth factor.

The CAM assay was introduced by Auerbach et al. in 1974 101, the corneal micropocket assay by Gimbrone et al. in 1974 102, the rat mesentery assay by Norrby et al. in 1986 68 [the assay has recently been demonstrated and discussed in a DVD movie with added detailed protocols in an Open Access journal 86], and the Matrigel assay was introduced by Passaniti et al. in 1992 103.

These analyses can also readily be performed using the mesentery.

Inbred and outbred mouse and rat strains are available.

Genetically engineered or immunocompromised mice are available.

As with all current angiogenesis assays, the release rate and the spatial and temporal distributions of exogenous pro- and anti-angiogenic test factors are not fully known, so uncontestable analyses of dose-effects are not feasible.

This enables for the first time the large-scale accurate counting and measurement of representative populations of individual sprouts and individual microvessel segments in any tissue.

Can be used for real-time observations following exteriorization of the mesentery; the model has been proven extremely useful in identifying novel cellular events in angiogenesis 57,58,104. However, the homeostasis of the tissue is rapidly disturbed outside the body, as discussed previously 70.

The rat mesentery angiogenesis assay (Table2) distinguishes itself from the other mammalian assays in that it is non-surgical (non-traumatic with minimal or no inflammation induced), the test tissue is naively vascularized (albeit sparsely), and it lacks physiologic angiogenesis in adulthood 70,86. This assay allows the quantitation (in exceptional detail) of angiogenesis with respect to a number of objective parameters, owing to the fact that the membranous test tissue is extremely thin and can be analyzed microscopically in intact form. Signaling events that are measured in the mesentery after stimulation with VEGF-A can be attributed with confidence to events that take place in the ECs 87. Moreover, when syngeneic ascitic tumor cells are transplanted into the mouse peritoneal cavity, the mesentery exhibits a complete repertoire of biological responses that can be attributed to VEGF-A, including sprouting angiogenesis 87.

### A case study: iron-unsaturated bovine lactoferrin inhibits angiogenesis in rats, mice, and chicken embryos, inhibits tumor angiogenesis, carcinogenesis, and metastasis in mice and rats, and it retards the growth of precancerous adenomatous colonic polyps in humans

Lactoferrin (LF) is an iron- and heparin-binding glycoprotein that is present in most biological fluids of mammals, including milk (colostral and mature), saliva, tears, and mucous, and it is released from activated neutrophils during inflammatory responses. The iron-unsaturated form of LF, termed apoLF (aLF), appears to be the dominant form of LF *in vivo*. ApoLF exerts antioxidant activity, is multifunctional and immunostimulatory, and exerts an antimicrobial activity thanks to a specific affinity for bacterial cell membranes 88. When LF becomes iron-saturated, remarkable changes occur in the 3-D structure of the molecule, its molecular flexibility and activities 89, including loss of antimicrobial action. Bovine and human LF have been shown to be non-toxic even after long-term oral administration to rats 90,91.

Bovine HαLF (abLF) and lactoferricin, a basic peptide of LF, although non-cytotoxic, inhibit the *in vitro* proliferation of human macrovascular ECs (HUVECs) in terms of both basal proliferation and proliferation in response to bFGF or VEGF165 92,93.

Metronomically scheduled ingestion of natural bovine HαLF (abLF) has been shown to suppress significantly VEGF-A-mediated angiogenesis in the rat mesentery assay 92. Angiogenesis in the CAM assay was inhibited by local application of abLF, whereas metronomic oral or intraperitoneal administration of abLF inhibited cancer cell-induced angiogenesis in a murine dorsal air sac model 94.

Following oral intake, pepsin degradation of LF yields lactoferricin, LFcin. Bovine lactoferricin, bLFcin, inhibits bFGF- and VEGF-A-induced angiogenesis in mice by competing for heparin-like binding sites on ECs, as assessed using the subcutaneous Matrigel plug assay 93. Systemic administration of abLF or bLFcin suppresses carcinogenesis in many organs, and inhibits metastasis formation in mice and rats 95–97. Metronomic ingestion of bLF by transgenic mice carrying the human VEGF-A165 gene, which spontaneously develop autochtonous pulmonary tumors, suppresses the expression of VEGF-A and tumor development 98. Moreover, the ingestion of HβLF (abLF) significantly delays the growth of precancerous adenomatous colonic polyps in patients 99.

These results confirm that the anti-angiogenic effects recorded in tumor-free models, such as the rat mesentery assay or the subcutaneous mouse Matrigel assay, can, under certain conditions, be valid for tumor angiogenesis and tumor growth suppression in several species, including humans.

## Concluding Remarks

Based on the available experimental and clinical data, it seems worthwhile to attempt to exploit the full potential of metronomic chemotherapy for clinical use. An initial requirement for reaching this goal is the introduction of biologically upgraded pre-clinical assays for both *in vitro* and *in vivo* analyses. These assays should have the ability to provide data that can be used with confidence to guide the design of more efficacious clinical trials. A second requirement is the introduction of reliable biomarkers for the assessment of anti-angiogenic responses in patients.

With respect to *in vitro* studies, it appears that reproducible and biologically more reliable results could be achieved by rectifying the culturing procedures that result in artifactual effects in the cells. Critical choices are needed to be made regarding the growth medium buffer, optimal control of the pH of the medium, and control of the oxygen pressure, which would reduce both the risk of super-physiologic intracellular ROS generation and the abnormal level of ROS in the medium. These adjustments could be achieved by employing techniques that already exist but which are not commonly employed collectively. An increased level of ROS, i.e., oxidative stress, activates numerous major signaling pathways and changes the gene expression profile of the cells. Adjustments along these lines in relation to *in vitro* procedures would facilitate comparisons between experiments and arguably strengthen the rationale for further animal and clinical studies. In particular, the issue of redox balance is bothersome when chemotherapeutic agents are involved, as these agents potently trigger ROS production, thereby further modifying the functions of the cultured cells. Co-cultures of ECs and other cell types, particularly perivascular cells and fibroblasts, are important in that they imitate the complex intercellular activities of angiogenesis *in vivo*.

Characteristics that, in our view, exemplify high-quality *in vivo* angiogenesis assays are listed in Table1. This list should probably be extended. Novel or upgraded assays lacking injury-induced wound healing and inflammatory responses, which blur results concerning anti-angiogenesis processes, are eagerly awaited. It is likely, we believe, that mammalian high-quality *in vivo* assays will generate biologically pertinent information, which will assist in the design of successful clinical trials.

Improved pre-clinical *in vivo* models might be helpful in identifying bona fide biomarkers that are useful for the appraisal of anti-angiogenic responses in patients. The challenging pre-clinical findings of strict drug-specific effects (anti-angiogenic or no effect on angiogenesis), and in several cases even pro-angiogenic effects, for standard cytotoxic drugs, as well as the antagonistic and unforeseeable effects of drug combinations following metronomic chemotherapy require further study. Although the experience from clinical trials speaks for the use of combination therapies, one may wonder if there is a need to re-examine the rationale behind metronomic combination drug therapies, which hitherto have not been especially successful in the clinic.

The recently reported effect of antioxidants on the angiogenesis-modulating outcome of metronomic chemotherapy, including the significance of the ‘metronomic chemotherapy vehicle factor’, is also a topic that needs further consideration.

As the focus is on the anti-angiogenic and consequential anti-tumor effects of metronomic chemotherapy, appropriate pre-clinical tumor models are required. The widely used syngeneic (non-orthotopic or orthotopic) and xenograft tumors are considered to be of questionable value, as they differ substantially in many critical biological respects from the majority of tumors seen in clinical practice. Spontaneously developing autochthonous tumors in genetically engineered mice, which exhibit lymphangiogenesis, an intact immune system, and unperturbed DNA repair mechanisms, seem to be considerably more appropriate models of human cancers, as they exhibit a natural stroma–tumor relationship. Moreover, these tumors allow important studies on the influence of stem cells on angiogenesis, and as they develop *de novo,* they are less artificial in terms of growth characteristics and genetic abnormalities than other currently used pre-clinical tumor models.

## References

[b1] Martindale JL, Holbrook NJ (2002). Cellular response to oxidative stress: signaling for suicide and survival. J Cell Physiol.

[b2] Gupta SC, Hevia D, Patchva S, Park B, Koh W, Aggarwal BB (2012). Upsides and downsides of reactive oxygen species for cancer: the roles of reactive oxygen species in tumorigenesis, prevention, and therapy. Antioxid Redox Signal.

[b3] Browder T, Butterfield CE, Kraling BM, Shi B, Marshall B, O’Reilly MS (2000). Antiangiogenic scheduling of chemotherapy improves efficacy against experimental drug-resistant cancer. Cancer Res.

[b4] Hanahan D, Bergers G, Bergsland E (2000). Less is more, regularly: metronomic dosing of cytotoxic drugs can target tumor angiogenesis in mice. J Clin Invest.

[b5] Klement G, Baruchel S, Rak J, Man S, Clark K, Hicklin DJ (2000). Continuous low-dose therapy with vinblastine and VEGF receptor-2 antibody induces sustained tumor regression without overt toxicity. J Clin Invest.

[b6] Weiss SM, Cheresh DA (2011). Tumor angiogenesis: molecular pathways and therapeutic targets. Nat Med.

[b7] Shaked Y, Bertolini F, Man S, Rogers MS, Cervi D, Foutz T (2005). Genetic heterogeneity of the vasculogenic phenotype parallels angiogenesis: implications for cellular surrogate marker analysis of antiangiogenesis. Cancer Cell.

[b8] Mancuso P, Colleoni M, Calleri A, Orlando L, Maisonneuve P, Pruneri G (2006). Circulating endothelial-cell kinetics and viability predict survival in breast cancer patients receiving metronomic chemotherapy. Blood.

[b9] Streubel B, Chott A, Huber D, Exner M, Jager U, Wagner O (2004). Lymphoma-specific genetic aberrations in microvascular endothelial cells in B-cell lymphomas. N Engl J Med.

[b10] Hida K, Hida Y, Shindoh M (2008). Understanding tumor cell abnormalities to develop ideal anti-angiogenic therapies. Cancer Sci.

[b11] Pasquier E, Kavallaris M, Andre N (2010). Metronomic chemotherapy: new rationale for new directions. Nat Rev.

[b12] Ricci-Vitiani L, Pallini R, Biffoni M, Todaro M, Invernici G, Cenci T (2010). Tumour vascularization via endothelial differentiation of glioblastoma stem-like cells. Nature.

[b13] Wang R, Chadalavada K, Wilshire J, Kowalik U, Hovinga KE, Geber A (2010). Glioblastoma stem-like cells give rise to tumour endothelium. Nature.

[b14] McGuire TF, Sajithlal GB, Lu J, Nicholls RD, Prochownik EV (2012). In vivo evolution of tumor-derived endothelial cells. PLoS ONE.

[b15] Sajithlal GB, McGuire TF, Lu J, Beer-Stolz D, Prochownik EV (2010). Endothelial-like cells derived directly from human tumor xenografts. Int J Cancer.

[b16] Deshane J, Chen S, Caballero S, Grochot-Przeczek A, Was H, Li Calzi S (2007). Stromal cell-derived factor 1 promotes angiogenesis via a hemen oxygenase 1-dependent mechanism. J Exp Med.

[b17] Newman AC, Chou W, Welch-Reardon KM, Fong AH, Popson SA, Phan DT (2013). Analysis of stromal cell secretomes reveals a critical role of stromal cell-derived hepatocyte growth factor and fibronectin in angiogenesis. Arterioscl Thromb Vasc Biol.

[b18] Bocci G, Francia G, Man S, Lawler J, Kerbel RS (2003). Thrombospondin 1, a mediator of the antiangiogenic effects of low-dose metronomic chemotherapy. PNAS.

[b19] Hamano Y, Sugimoto H, Soubasakos MA, Kieran M, Olsen BR, Lawler J (2004). Thrombospondin-1 associated with tumor microenvironment contributes to low-dose cyclophosphamide-mediated endothelial cell apoptosis and tumor growth suppression. Cancer Res.

[b20] Damber JE, Vallbo C, Albertsson P, Lennernas B, Norrby K (2006). The anti-tumor effect of low-dose continuous chemotherapy may partly be mediated by thrombospondin. Cancer Chem Pharmacol.

[b21] Pasquier E, Kieran MW, Sterba J, Shaked Y, Baruchel S, Oberlin O (2011). Moving forward with metronomic chemotherapy: meeting report of the 2nd International Workshop on Metronomic and Anti-Angiogenic Chemotherapy in Paediatric Oncology. Transl Oncol.

[b22] Kerbel RS, Kamen BA (2004). The anti-angiogenic basis of metronomic chemotherapy. Nat Rev.

[b23] Dellapasqua S, Bertolini F, Banardi V, Campagnoli E, Scarano E, Torrisi R (2008). Metronomic cyclophosphamide and capecitabine combined with bevacizumab in advanced breast cancer. J Clin Oncol.

[b24] Calleri A, Bono A, Bagnardi V, Quarna J, Mancuso P, Rabascio C (2009). Predictive potential of angiogenic growth factors and circulating endothelial cells in breast cancer patients receiving metronomic chemotherapy plus bevacizumab. Clin Cancer Res.

[b25] Sikder H, Huso DL, Zhang H, Wang B, Ryu B, Hwang ST (2003). Disruption of Id1 reveals major differences in angiogenesis between transplanted and autochthonous tumors. Cancer Cell.

[b26] Emmenegger U, Kerbel RS (2007). Five years of clinical experience with metronomic chemotherapy: achievements and perspectives. Onkologie.

[b27] Reynolds AR (2010). Potential relevance of Bell-shaped and U-shaped dose-responses for the therapeutic targeting of angiogenesis in cancer. Dose-Response.

[b28] Staton CA, Reed MWR, Brown NJ (2009). A critical analysis of current in vitro and in vivo angiogenesis assays. Int J Exp Path.

[b29] Albertsson P, Lennernas B, Norrby K (2006). On metronomic chemotherapy: modulation of angiogenesis mediated by VEGF-A. Acta Oncol.

[b30] Albertsson P, Lennernas B, Norrby K (2008). Dose effects of continuous vinblastine chemotherapy on mammalian angiogenesis mediated by VEGF-A. Acta Oncol.

[b31] Albertsson P, Lennernas B, Norrby K (2009). Low-dose continuous 5-fluorouracil infusion stimulates VEGF-A-mediated angiogenesis. Acta Oncol.

[b32] Albertsson P, Lennernas B, Norrby K (2012). Low-dose metronomic chemotherapy and angiogenesis: topoisomerase inhibitors irinotecan and mitoxantrone stimulate VEGF-A-mediated angiogenesis. APMIS.

[b33] Norrby K, Nordenhem A (2010). Dalteparin, a low-molecular-weight heparin, promotes angiogenesis mediated by heparin-binding VEGF-A in vivo. APMIS.

[b34] Albertsson P, Lennernas B, Norrby K (2003). Chemotherapy and antiangiogenesis: drug-specific effects on microvessel sprouting. APMIS.

[b35] Lennernas B, Albertsson P, Lennernas H, Norrby K (2003). Chemotherapy and antiangiogenesis: drug-specific, dose-related effects. Acta Oncol.

[b36] Fioravanti A, Canu B, Ali G, Orlandi P, Allegrini G, Di Desidero T (2009). Metronomic 5-fluorouracil, oxaliplatin and irinotecan in colorectal cancer. Eur J Pharmacol.

[b37] Halliwell B (2008). Are polyphenols antioxidants or pro-oxidants? What do we learn from cell culture and in vivo studies. Arch Biochem Biophys.

[b38] Finkel T, Holbrook N (2000). Oxidants, oxidative stress and the biology of ageing. Nature.

[b39] Finkel T (2003). Oxidant signals and oxidative stress. Curr Opin Cell Biol.

[b40] Uy B, McGlashan SR, Shaikh SB (2011). Measurement of reactive oxygen species in the culture media using Acridan Lumigen PS-3 assay. J Biomol Tech.

[b41] Grzelak A, Rychlik B, Bartosz G (2000). Reactive oxygen species are formed in cell media. Acta Biochim Pol.

[b42] Maguire A, Morrissey B, Walsh JE, Lyng FM (2011). Medium-mediated effects increase cell killing in a human keratinocyte cell line exposed to solar-stimulated radiation. Int J Radiat Biol.

[b43] Pevec AZ, Slejkovec Z, van Elteren JT, Falnoga I (2012). As_2_O_3_ oxidation by vitamin C: cell culture studies. Biometals.

[b44] Lewinska A, Wnuk M, Slota E, Bartosz G (2007). Total anti-oxidant capacity of cell culture media. Clin Exp Pharmacol Physiol.

[b45] Dröge W (2002). Free radicals in the physiological control of cell function. Physiol Rev.

[b46] Kuroki M, Voest EE, Amano S, Beerepoot LV, Takashima S, Tolentino M (1996). Reactive oxygen intermediates increases vascular endothelial growth factor expression in vitro and in vivo. J Clin Invest.

[b47] Gerber H-P, McMurtrey A, Koalski J, Yan M, Keyt BA, Dixit V (1998). Vascular endothelial growth factor regulates endothelial cell survival through phosphatidylinositol 3′-kinase/Akt signal transduction pathway. J Biol Chem.

[b48] Colavitti R, Pani G, Bedogni B, Anzevino R, Borrello S, Waltenberger J (2002). Reactive oxygen species as downstream mediators of angiogenic signaling by vascular endothelial growth factor receptor-2/kdr. J Biol Chem.

[b49] Maulik N, Das DK (2002). Redox signaling in vascular angiogenesis. Free Rad Biol Med.

[b50] Patra CR, Kim J-H, Pramanik K, d’Uscio LV, Patra S, Pal K (2011). Reactive oxygen species driven angiogenesis by inorganic nanorods. Nano Lett.

[b51] Li Y, Rory Goodwin C, Sang Y, Rosen EM, Laterra J, Xia S (2009). Camptothecin and Fas receptor agonists synergistically induce medulloblastoma cell death: ROS-dependent mechanisms. Anti Cancer Drugs.

[b52] Yamada T, Egashira N, Imuta M, Yano T, Yamauchi Y, Watanabe H (2010). Role of oxidative stress in vinorelbine-induced vascular endothelial cell injury. Free Rad Biol Med.

[b53] Liu F, Smith J, Zhang Z, Cole R, Herron BJ (2010). Genetic heterogeneity of skin microvasculature. Dev Biol.

[b54] Lau ATY, Wang Y, Chiu J-F (2008). Reactive oxygen species: current knowledge and applications in cancer research and therapeutic. J Cell Biochem.

[b55] Verbridge SS, Choi NW, Zheng Y, Brooks DJ, Stoock AD, Fischbach C (2010). Oxygen-controlled three-dimensional cultures to analyze tumor angiogenesis. Tissue Eng Part A.

[b56] Yang M, Stapor PC, Peirce SM, Betancourt AM, Murfee WL (2012). Rat mesentery exteriorization: a model for investigating the cellular dynamics involved in angiogenesis. J Vis Exp.

[b57] Stapor PC, Azimi MS, Ahsan T, Murfee WL (2013). An angiogenesis model for investigating multicellular interactions across intact microvascular networks. Am J Physiol Heart Circ Physiol.

[b58] Mantovani A, Allavena P, Sica A, Balkwill F (2008). Cancer-related inflammation. Nature.

[b59] Norrby K, Enerbäck L, Franzén L (1976). Mast cell activation and tissue cell proliferation. Cell Tiss Res.

[b60] Norrby K, Enestrom S (1984). Cellular and extracellular changes following mast-cell secretion in avascular rat mesentery. An electron-microscopic study. Cell Tiss Res.

[b61] Norrby K (2002). Mast cells and angiogenesis. APMIS.

[b62] Fainaru O, Adini A, Benny O, Adini I, Short S, Bazinet L (2008). Dendritic cells support angiogenesis and promote lesion growth in a murine model of endometriosis. FASEB J.

[b63] Lin EY, Pollard JW (2007). Tumor-associated macrophages press the switch in breast cancer. Cancer Res.

[b64] Sozzani S, Rusnati M, Riboldi E, Mitola S, Presta M (2007). Dendritic cell-endothelial cross-talk in angiogenesis. Trends Immunol.

[b65] Fainaru O, Almog N, Yung CW, Nakai K, Montoya-Zavala M, Abdollahi A (2010). Tumor growth and angiogenesis are dependent on the presence of immature dendritic cells. FASEB J.

[b66] Coussens LM, Werb Z (2002). Inflammation and cancer. Nature.

[b67] Soucek L, Lawlor ER, Soto D, Shchors K, Swigart LB, Evan GI (2007). Mast cells are required for angiogenesis and macroscopic expansion of Myc-induced pancreatic islet tumors. Nat Med.

[b68] Norrby K, Jakobsson A, Sörbo J (1986). Mast-cell-mediated angiogenesis: a novel experimental model using the rat mesentery. Virchows Arch B Cell Pathol Incl Mol Pathol.

[b69] Kinet J-P (2007). The essential role of mast cells in orchestrating inflammation. Immunol Rev.

[b70] Norrby K (2006). In vivo models of angiogenesis. J Cell Mol Med.

[b71] Yang Y, Zhang Y, Hong H, Liu G, Leigh BR, Cai W (2011). In vivo near-infrared fluorescence imaging of CD105 expression during tumor angiogenesis. Eur J Nucl Med Imaging.

[b72] Hlatky L, Hahnfeldt P, Folkman J (2002). Clinical application of antiangiogenic therapy: microvessel density, what it does and doesn’t tell us. J Natl Cancer Inst.

[b73] Hendrix MJ, Seftor EA, Seftor RE, Kasemeier-Kulesa J, Kulesa PM, Postovit LM (2007). Reprogramming metastatic tumour cells with embryonic microenvironments. Nat Rev Cancer.

[b74] Olive KP, Jacobetz MA, Davidson CJ, Gopinathan A, McIntyre D, Honess D (2009). Inhibition of Hedgehog signaling enhances delivery of chemotherapy in a mouse model of pancreatic cancer. Science.

[b75] Combest AJ, Roberts PJ, Dillon PM, Sandison K, Hanna SK, Ross C (2012). Genetically engineered cancer models, but not xenografts, faithfully predict anticancer drug exposure in melanoma tumors. Oncologist.

[b76] Rohan RM, Fernandez A, Udagawa T, Yuan J, D’Amato RJ (2000). Genetic heterogeneity of angiogenesis in mice. FASEB J.

[b77] Sampaio FP, Castro PR, Marques SM, Campos PP, Ferreira MA, Andrade SP (2012). Genetic background determines inflammatory angiogenesis response to dipyridamole in mice. Exp Biol Med.

[b78] Marques SM, Campos PP, Castro PR, Cardoso CC, Ferreira MA, Andrade SP (2011). Genetic background determines mouse strain differences in inflammatory angiogenesis. Microvasc Res.

[b79] Italiano JE, Richardson JL, Patel-Hett S, Battinelli E, Zaslavsky A, Short S (2008). Angiogenesis is regulated by a novel mechanism: pro- and antiangiogenic proteins are organized into separate platelet (alpha) granules and differentially released. Blood.

[b80] Battinelli EM, Markens BA, Italiano JE (2011). Release of angiogenesis regulatory proteins from platelet alpha granules: modulation of physiologic and pathologic angiogenesis. Blood.

[b81] Sabrkhany S, Griffioen AW, oude Egbrink MGA (2011). The role of blood platelets in tumor angiogenesis. Biochim Biophys Acta.

[b82] St. Croix B, Rago C, Velculescu V, Traverso G, Romans KE, Montgomery E (2000). Genes expressed in human tumor endothelium. Science.

[b83] Hasan J, Shnyder SD, Bibby M, Double JA, Bicknell R, Jayson GC (2004). Quantitative angiogenesis assays in vivo–a review. Angiogenesis.

[b84] Auerbach R, Staton CA, Lewis C, Bicknell R (2006). An Overview of Current Angiogenesis Assays: Choice of Assay, Precautions in Interpretation, Future Requirements and Directions. Angiogenesis Assays.

[b85] Melero-Martin JM, Bischoff J (2008). An in vivo experimental model for postnatal vasculogenesis. Meth Enzymol.

[b86] Norrby KC (2011). Rat mesentery angiogenesis assay. J Vis Exp.

[b87] Mukhopadhyay D, Nagy JA, Manseau EJ, Dvorak HF (1998). Vascular permeability factor/vascular growth factor-mediated signaling in mouse mesentery vascular endothelium. Cancer Res.

[b88] Bellamy W, Takase M, Yamauchi K, Wakabayashi H, Kawase K, Tomita M (1992). Identification of the bactericidal domain of lactoferrin. Biochim Biophys Acta.

[b89] Baker EN, Anderson BF, Baker HM, Haridas M, Jameson GB, Norris GE (1991). Structure, function and flexibility of human lactoferrin. Int J Biol Macromol.

[b90] Cerven D, DeGeorge G, Bethell D (2008). 28-day repeated dose oral toxicity of recombinant human apo-lactoferrin or recombinant human lysozyme in rats. Reg Tox Pharmacol.

[b91] Tamano S, Sekine K, Takase M, Yamauchi K, Iigo M, Tsuda H (2008). Lack of chronic oral toxicity of chemopreventive bovine lactoferrin in F344/DuCrj rats. Asian Pac J Cancer Prev.

[b92] Norrby K, Mattsby-Baltzer I, Innocenti M, Tuneberg S (2001). Orally administered bovine lactoferrin systemically inhibits VEGF165-mediated angiogenesis in the rat. Int J Cancer.

[b93] Mader JS, Smyth D, Marshall J, Hoskin DW (2006). Bovine lactoferricin inhibits fibroblast growth factor- and vascular endothelial growth factor165-induced angiogenesis by competing for heparin-like binding sites on endothelial cells. Am J Pathol.

[b94] Shimamura M, Yamamoto Y, Ashino H, Oikawa T, Hazato T, Tsuda H (2004). Bovine lactoferrin inhibits tumor-induced angiogenesis. Int J Cancer.

[b95] Sekine K, Watanabe E, Nakamura J, Takasuka N, Kim DJ, Asamoto M (1967). Inhibition of Azoxymethane-initiated colon tumour by bovine lactoferrin administration in F344 rats. Jpn J Cancer Res.

[b96] Yoo YC, Watanabe S, Watanabe R, Hata K, Shimazaki K, Azuma I (1997). Bovine lactoferrin and lactoferricin, a peptide derived from bovine lactoferrin, inhibit tumor metastasis in mice. Jpn J Cancer Res.

[b97] Tsuda H, Kozu T, Iinuma G, Ohashi Y, Saito Y, Akasu T (2010). Cancer prevention by bovine lactoferrin: from animal studies to human trial. Biometals.

[b98] Tung YT, Chen HL, Yen CC, Lee PY, Tsai HC, Lin MF (2013). Bovine lactoferrin inhibits lung cancer growth through suppression of both inflammation and expression of vascular endothelial growth factor. J Dairy Sci.

[b99] Kozu T, Iinuma G, Ohashi Y, Saito Y, Akasu T, Saito D (2009). Effect of orally administered bovine lactoferrin on the growth of adenomatous colorectal polyps in a randomized, placebo-controlled clinical trial. Cancer Prev Res.

[b100] Adini A, Fainaru O, Udagawa T, Connor KM, Folkman J, D’Amato RJ (2009). Matrigel cytometry: a novel method for quantifying angiogenesis in vivo. J Immunol Methods.

[b101] Auerbach R, Kubai L, Knighton D, Folkman J (1974). A simple procedure for the long-term cultivation of chicken embryos. Dev Biol.

[b102] Gimbrone MA, Cotran RS, Leapman SB, Folkman J (1974). Tumor growth and neovascularization: an experimental model using the rabbit cornea. J Natl Cancer Inst.

[b103] Passaniti A, Taylor RM, Pili R, Guo Y, Long PV, Haney JA (1992). A simple, quantitative method for assessing angiogenesis and antiangiogenic agents using reconstituted basement membrane, heparin, and fibroblast growth factor. Lab Invest.

[b104] Anderson CR, Hastings NE, Blackman BR, Price RJ (2008). Capillary sprout endothelial cells exhibit a CD36 low phenotype: regulation by shear stress and vascular endothelial growth factor-induced mechanism for attenuating anti-proliferative thrombospondin-1 signaling. Am J Pathol.

[b105] Mattsby-Baltzer I, Jakobsson A, Sorbo J, Norrby K (1994). Endotoxin is angiogenic. Int J Exp Path.

[b106] Ramanathan M, Pinhal-Enfield G, Hao I, Leibovich SJ (2007). Synergistic up-regulation of vascular endothelial growth factor (VEGF) expression in macrophages by adenosine A2A receptor agonists and endotoxin involves transcriptional regulation via the hypoxia response element in the VEGF promoter. Mol Biol Cell.

